# Is there any association between dietary inflammatory index and quality of life? A systematic review

**DOI:** 10.3389/fnut.2022.1067468

**Published:** 2022-12-22

**Authors:** Mona Golmohammadi, Sorayya Kheirouri, Vahideh Ebrahimzadeh Attari, Jalal Moludi, Reny Sulistyowati, Seyed Mostafa Nachvak, Roghayeh Mostafaei, Maryam Mansordehghan

**Affiliations:** ^1^Department of Nutritional Sciences, School of Nutritional Sciences and Food Technology, Kermanshah University of Medical Sciences, Kermanshah, Iran; ^2^Department of Community Medicine, School of Nutrition and Food Sciences, Tabriz University of Medical Sciences, Tabriz, Iran; ^3^Department of Nutrition and Food Sciences, Maragheh University of Medical Sciences, Maragheh, Iran; ^4^Poltekkes Kemenkes Palangka Raya, Palangka Raya, Indonesia

**Keywords:** anti-inflammatory diet, chronic disease, dietary inflammatory index, inflammation, quality of life

## Abstract

**Background:**

The inflammatory potential of unhealthy diets can lead to the development of chronic diseases and also exacerbating their complications. Therefore, the present systematic review aimed to evaluate the association of dietary inflammatory index (DII) and quality of life (QOL) in human subjects.

**Methods:**

A systematic search was conducted in PubMed, Web of Science, and Scopus databases, using the combination of all search terms related to DII and QOL until May 2022. All eligible human studies published in English were included.

**Results:**

Three hundred twenty-seven studies were obtained from the first systematic search of the databases although, only eight studies were eligible for the evaluation. Seven studies reported that there was a significant reverse association between DII scores and overall QOL and/or its subscales in different populations including patients with asthma, osteoarthritis, hemodialysis patients, multiple sclerosis, obese women, and also in healthy subjects. While, one study on postmenopausal women found no evidence of this association.

**Conclusion:**

This systematic review demonstrated that an anti-inflammatory diet might be associated with better QOL. However, future well-designed clinical trials can provide better conclusions especially regarding the quantifying of this relationship.

## Introduction

The term Quality of Life (QOL) was first introduced in 1970s, as the multi-dimensional concept of well-being and health status regarding the physical, mental, emotional, and social aspects of life (1). QOL usually decreases during the aging and diseases (2, 3). It has been documented that low-grade inflammation, increasing pro-inflammatory cytokines in the body, is associated with different chronic disease (4–9), as well as impaired neurodevelopment (10) and adverse mental health outcomes (11), which can affect various aspects of patients’ QOL (12). Therefore, reversing the inflammatory pathways can increase QOL of patients.

Emerging evidence showed that healthy eating is associated with low inflammatory responses and can be a cost-effective intervention to improve the QOL. It was reported that a Western dietary pattern with high consumption of refined grains, processed meats, butter, and high-fat dairy products causes inflammation in the body. Whereas, a healthy diet like the Mediterranean diet which includes whole grains, vegetables, fish, and olive oil, can prevent inflammation or suppress inflammatory pathways (13–16). For this purpose, Shivappa et al. developed a tool to assess the inflammatory potential of the diet called the Dietary Inflammatory Index (DII) (17). Higher DII scores are associated with inflammatory cytokines such as interleukin (IL)-6, tumor necrosis factor (TNF-α), and high-sensitivity C-reactive protein (hs-CRP) (18, 19). Studies have shown that DII is associated with various diseases such as breast cancer (20), colorectal cancer (21), osteoarthritis (22), metabolic syndrome (23), and asthma (24).

Therefore, this study was conducted with the hypothesis that the inflammatory potential of the diet can lead to the development or progression of chronic diseases complications and thus decreases patients’ QOL. To the best of our knowledge, this is the first systematic review that has summarized and concluded the outcomes of related studies to assess the impact of DII on QOL.

## Methods

### The search strategy

This study was performed according to the PRISMA-P (Preferred Reporting Items for Systematic Reviews and Meta-

Analyses Protocols) 2015 statement. We searched through PubMed/Medline, Web of Science, and Scopus for relevant papers published in English until May 2022 using the following keywords: “dietary inflammatory score” OR “dietary inflammatory index” OR “DII” OR “inflammatory diet” OR “inflammatory potential of diet” OR “dietary inflammation potential” OR “potential inflammatory intake” OR “anti-inflammatory diet” OR “pro-inflammatory diet” in title/abstract AND “quality of life” OR “QOL” OR “health-related QOL” OR “HRQOL” OR “World Health Organization Quality of Life-Brief” OR “WHOQOL” OR “PedsQL” in the title/abstract.

### The screening of studies

All detected articles were saved in an EndNote software file and duplicate articles were removed. Then, unrelated articles were identified and deleted by reviewing the titles and abstracts. The full text of remaining articles was then screened for eligibility and data extraction by two independent researchers. Discrepancies between the two authors were resolved by a third researcher.

### Inclusion and exclusion criteria

Studies were included if they examined the association of a DII score and QOL. There was no restriction on study design and all English articles were eligible. Moreover, studies that assessed the association of DII with QOL in patients with knee osteoarthritis, multiple sclerosis (MS), asthma, and hemodialysis were included in this review.

### Data extraction and quality assessment

The data were collected according to a standard extraction form to obtain the information about the first author’s name, geographical area, study design, population/sample size, mean ages of participants, interventional/control diet, duration of intervention, QOL/DII/food intake assessment tools, and the main outcomes.

For assessment of the articles quality, the adapted version of the Newcastle–Ottawa Scale (NOS) checklist was used for cross-sectional studies as it was shown in Supplementary Table 1 (25) and the Jadad checklist was used for experimental studies as it was shown in Supplementary Table 2 (26). In the NOS checklist, the score of ≥7 was interpreted as a low risk of bias, scores between 4 and 6 were interpreted as a high risk of bias, and the score of <4 was interpreted as a very high risk of bias (27). In the Jadad checklist, the score of ≥3 was considered to have superior quality (26).

## Results

### Selection of studies

**FIGURE 1:**
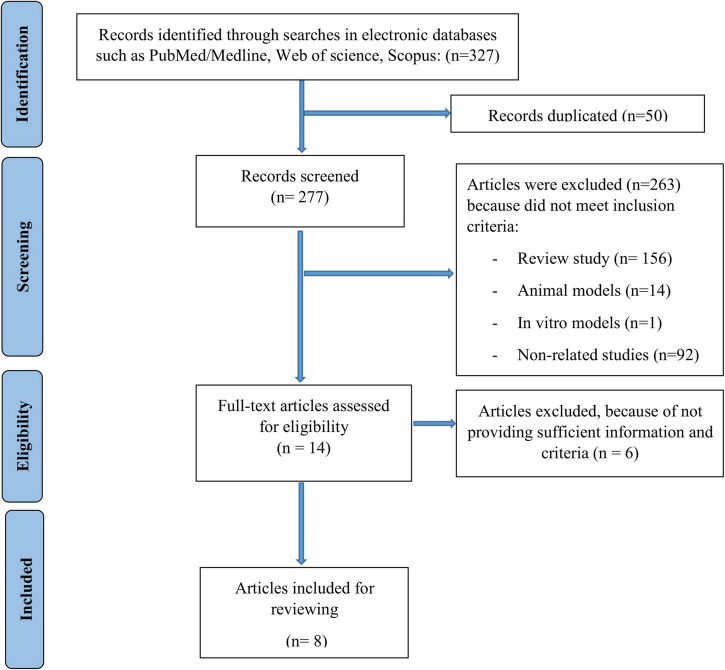
Flowchart of the studies search and selection.

As it was shown in Figure 1, 327 potentially relevant articles were obtained by the search strategy. Of these records, 50 were excluded due to duplicate studies. Then, of 277 remained articles, 263 studies were excluded because they did not meet the inclusion criteria. Finally, 8 articles were included for analysis.

### Characteristics of included studies

The study population of included studies were as follow: knee osteoarthritis (*n* = 1) (28), asthma (*n* = 1) (29), MS (*n* = 1) (30), hemodialysis patients (*n* = 1) (31), women with obesity or overweight (*n* = 1) (32), postmenopausal women (*n* = 1) (33), healthy people (*n* = 2) (34, 35). The details of each study are summarized in Table 1.

**TABLE 1:** Summary of included studies.

References, country	Type of study	Population/ Sample size	Age (year)	Interventional diet	Control diet	Duration of intervention (weeks)	Quality of life assessment tools	DII assessment method	Food intake assessment tools	Main outcomes
Lycett et al. (35), Australia	Cross-sectional	Children *n* = 1,759 and adults *n* = 1,812	11.5 ± 0.5 and 43.7 ± 5.2	–	–	–	Child and adult version of the Child Health Utility-9D	26 food parameters	60 items FFQ	Higher DII scores were associated with lower QOL.
Song et al. (33), Korean	Cross-sectional	Postmenopausal women *n* = 132	45–70	–	–	–	EQ-5D	38 food parameters	3-day food record	QOL did not show a significant difference across the DII tertiles.
Kuczmarski et al. (34), USA	Cross-sectional	Urban African American and White adults *n* = 1,907	48.38 ± 0.21	–	–	–	SF-12	35 food parameters	4-day 24-h dietary recalls	Higher DII scores were associated with lower QOL.
Yaseri et al. (31), Iran	Cross-sectional	Hemodialysis patients *n* = 83	56.7 ± 12.6	–	–	–	SF-36	45 food parameters	3-day 24-h dietary recalls	Higher DII scores were associated with lower QOL.
Tabrizi et al. (32), Iran	Cross-sectional	Reproductive-aged women with obesity or overweight *n* = 278	31.40 ± 10.89	–	–	–	SF-36	24 food parameters	168 items FFQ	Higher DII scores were associated with lower QOL.
Toopchizadeh et al. (28), Iran	Cross-sectional	Knee osteoarthritis patients *n* = 220	≥45	–	–	–	SF-36	29 food parameters	168 items FFQ	Highest DII score was associated with lower QOL in terms of physical function, role limitation due to physical health, social function, and pain scales and physical health subscale.
Yucel et al. (29), Turkey	RCT	Obese asthmatic patients Intervention (*n* = 29) Control (*n* = 26)	Intervention.: 50.4 ± 10.4 Control.: 50.3 ± 10.0	–	No dietary recommendation	10 weeks	AQLQ	24 food parameters	2-day 24-h dietary recalls	AQLQ scores increased in the intervention group.
Mousavi-Shirazi-Fard et al. (30), Iran	RCT	Relapsing-remitting MS patients Intervention (*n* = 50) Control (*n* = 50)	Intervention: 35.20 ± 6.61 Control: 36.26 ± 7.23	Anti-inflammatory diet	Healthy diet	12 weeks	MSQOL-54	35 food parameters	147 items FFQ	Physical and mental components of MSQOL-54 was improved between and within the two groups after the intervention.

AQLQ, asthma quality of life questionnaire; DII, dietary inflammatory index; EQ-5D, EuroQOL-5D; FFQ, food frequency questionnaire; MS, multiple sclerosis; MSQOL-54, multiple sclerosis quality of life; QOL, quality of life; RCT, randomized control trial; SF-12, short-form 12; SF-36, short-form 36.

The population of studies were over 20 years of age, except for one research on children aged 11–12 (35). Of 8 included studies, 2 were randomized controlled trial (RCT) using the anti-inflammatory diets as the intervention for 10 (29) and 12 (30) weeks (see Table 1) and 6 articles were cross-sectional in design (28, 31–35).

Different questionnaires were used to assess QOL across the studies including Child and adult version of the Child Health Utility-9D (35), Short-Form 36 (SF-36) (28, 31, 32), Short-Form 12 (SF-12) (34), EuroQOL-5D (EQ-5D) (33), Asthma quality of life questionnaire (AQLQ) (29) and Multiple Sclerosis Quality of Life (MSQOL-54) (30).

There was also a heterogeneity in the assessment of dietary inflammatory index between studies. The food intake assessment tools were food frequency questionnaire (FFQ) (28, 30, 32, 35), 3-day food record (33), 3-day or 2-day 24-hour food recall (29, 31, 34).

### Quality of the articles

Using the NOS checklist, it was determined that four cross-sectional studies had a high risk of bias (28, 31–33) and two of them had a low risk of bias (34, 35). Jadad’s checklist also showed that all interventional studies had superior quality (29, 30). The scores obtained from the NOS and Jadad checklists are shown in Supplementary Tables 1, 2.

### Association between the dietary inflammatory index and quality of life

Five out of six cross-sectional studies found that higher DII scores were significantly associated with lower QOL (28, 31, 32, 34, 35). But the results of one cross-sectional study on post-menopausal women did not show any significant differences in QOL across the DII tertiles (33).

Moreover, the results of clinical trials showed that consumption of anti-inflammatory diet for 10 and 12 weeks significantly increased patients’ quality of life in terms of different physical and/or mental components (29, 30).

## Discussion

To the best of our knowledge, the present study is the first systematic review of the association between DII and QOL. The majority of the studies included in this review showed the negative relationship between DII with QOL and/or its domains, with the exception of one study showing no association (33).

Healthy dietary patterns such as the Mediterranean diet and the Dietary Approaches to Stop Hypertension (DASH) promote eating healthy foods, which can reduce inflammation in the body (36–39), while Western dietary pattern has inflammatory properties (40). The effect of dietary patterns on QOL have also been previously studied and our results are consistent with these studies. Results of a systematic review by Govindaraju et al. showed that healthy dietary patterns like Mediterranean diet were associated with better QOL (41). Another review study found that in contrast to the Western and unhealthy diet, the Mediterranean diet was associated with better QOL in both physical and mental domains (42). Wu et al. reported that diet quality and dietary behavior were positively associated with various aspects of QOL, including physical, psychosocial, school, and emotional functioning in children and adolescents (43). Results of a most recent study showed that adherence to the Mediterranean diet was positively associated with quality of life in children and adolescents (44). Moreover, adhering to the DASH pattern led to the improvement of QOL in patients with heart failure (HF) during 3 months (45). It was also reported that healthy dietary patterns were associated with better sleep status, sexual function, and physical activity (46–50).

The most important mechanism for the health effects of the aforementioned healthy dietary patterns can be justified by reducing inflammation, suppressing pro-inflammatory responses, and the antioxidant effects. In this regard, focusing on the effect of diet in modulation of inflammation caused to the development of DII first in 2009 (51). DII is a validated dietary score that was introduced to assess the potential effects of people’s diet on their inflammatory status and health outcomes. Accordingly, a high DII score reflects the pro-inflammatory potential of diet, while the low scores of DII reflect the anti-inflammatory effect of diet (52).

It was reported that high DII scores were positively associated with systemic inflammation and also decreased lung function in people with asthma (24). Moreover, it was reported that consumption of a pro-inflammatory diet may have important role in knee osteoarthritis pathology (53). Studies showed a positive association between DII scores with postmenopausal complications such as osteoarthritis (33), lower bone density (54, 55), higher menopause-specific somatic score (56), hip fracture risk (54), increased risk of breast cancer (20), and proximal colorectal cancer (21).

Bohlouli et al. showed that adherence to an anti-inflammatory diet such as the Mediterranean diet improved fatigue severity in relapsing-remitting MS (57). Cross-sectional studies showed that the body composition and anthropometric measurements were directly associated with DII scores (58, 59). There are evidence that high DII scores have been positively associated with an increased risk of obesity in non-obese individuals and also the prevalence of overweight and obesity (60). Recently, Ferreira et al. showed that the comorbidities of obesity decreased after improving the DII scores of participants (61).

Dietary inflammatory index can trigger inflammatory responses in the body (24, 62) and the inflammatory cytokines are related to low QOL due to physical disability, psychosocial burdens, pain, mood, and sexual function (63–72) in different conditions and diseases like respiratory tract diseases (24, 73–75), osteoarthritis and synovitis (76, 77), MS (78), obesity (79, 80), postmenopausal women (81, 82), and hemodialysis patients (83).

However, Song et al. showed that there was no significant relationship between DII scores and QOL in post-menopausal women, which may be due to the low sample size of the study (33).

**FIGURE 2:**
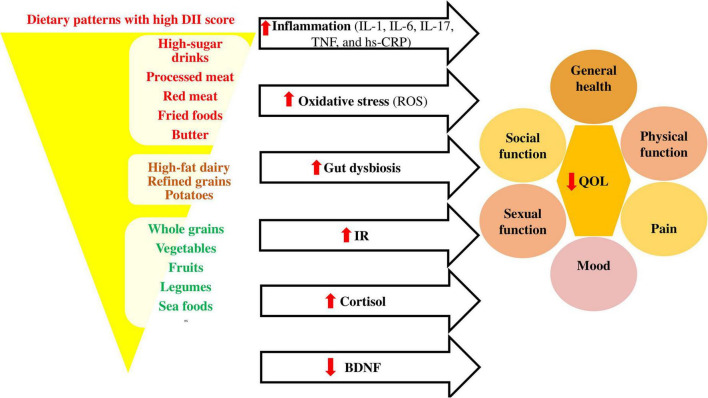
Association between pro-inflammatory diets and quality of life. Pro-inflammatory diets like Western diets (98) affect the inflammatory cytokines (99, 100), oxidative stress (101), gut microbiota composition (102), insulin resistance (IR) (103), cortisol (104), and brain derived neurotropic factor (BDNF) (105) levels, causing decrease in quality of life through its effects on different dimensions. BDNF, brain derived neurotropic factor; DII, dietary inflammatory index; hs-CRP, high-sensitivity C-reactive protein; IL, interleukin; IR, insulin resistance; QOL, quality of life; ROS, reactive oxygen species; TNF, tumor necrosis factor.

Figure 2 shows the association between pro-inflammatory diets and quality of life in different conditions.

Healthy dietary patterns with low DII scores can also change the gut microbiota composition and correct the gut dysbiosis (84). These diets emphasize the consumption of vegetables, fruits, whole grain, beans, legumes, nuts, seeds, and olive oil (85) and improve the microbiome diversity by increasing growth of Bacteroides, Lactobacili, Bifidobacteria, Faecalibacterium, Oscillospira, Roseburia, Ruminococci, and their metabolic activities and decreasing growth of Firmicutes and Proteobacteria (86). Therefore, the production of short-chain fatty acids (SCFAs) will increase in the feces (84). SCFAs, especially butyrate bind to epithelial and immune cell G protein-coupled receptors (GPCRs) which leads to maintaining the integrity of the intestine and preventing inflammation, oxidative stress, and insulin resistance (87), while the western diets lead to metabolic endotoxemia by increasing intestinal permeability (86). Indeed, gut dysbiosis can affect various aspects of QOL, including physical and mental health (88–92).

The antidepressant effect of healthy diets with low DII score can also be explained through decreasing cortisol (93) and increasing brain derived neurotropic factor (BDNF) (94–97). Several limitations in the present study should be clarified when interpreting the results of this review including: (a) The number of studies on the association of DII and QOL was limited. (b) There was heterogeneity between studies’ populations (different diseases or conditions) and also questionnaires which assessed the QOL, DII, and food intake. (c) The majority of included studies in this systematic review were cross-sectional studies, which did not show causal and temporal associations. (d) The instruments used to examine diet and quality of life were both self-reported, which may be subject to recall and reporting biases.

## Conclusion

This systematic review demonstrated that an anti-inflammatory diet might be associated with better QOL. However, future well-designed clinical trials on various disease can provide better conclusions especially regarding the quantifying of this relationship.

## Data availability statement

The original contributions presented in this study are included in this article/Supplementary material, further inquiries can be directed to the corresponding author.

## Author contributions

MG and SN contributed to designing the study, searching for resources, and writing the manuscript. MG, SK, VE, and JM cooperated in writing the manuscript. RS contributed to English revising of the manuscript. RM and MM cooperated in literature search. All authors contributed to the article and approved the submitted version.
