# Supplementary material for: Is there any association between dietary inflammatory index and quality of life? A systematic review
Source: Front Nutr. 2022 Dec 22;9:1067468. doi: 10.3389/fnut.2022.1067468 (PMC9815464; doi:10.3389/fnut.2022.1067468)
Supplement: Supplementary file 1 [file Data_Sheet_1.PDF]

Supplementary Material

1 Supplementary Tables

Supplemental Table 1. The adapted version of the Newcastle–Ottawa Scale (NOS) Checklist for Cross-Sectional studies

**Author, publication year**	**Study design**	**Selection**				**Comparability**	**Outcome**			
		**Representativeness of the sample**	**Sample size**	**Nonrespondents**	**Ascertainment of exposure**	**Based on design and analysis**	**Assessment of outcome**	**Statistical test**	**Score**	**Interpretation**
Lycett et al, 2022 (Lycett et al., 2022)	Cross-sectional	+		+	++	++	+		7	**Low risk of bias**
Song, et al, 2022 (Song et al., 2022)	Cross-sectional				++	++	+		5	High risk of bias
Kuczmarski et al, 2021 (Kuczmarski et al., 2021)	Cross-sectional	+	+	+	++	++	+		8	**Low risk of bias**
Yaseri et al, 2021 (Yaseri et al., 2021)	Cross-sectional				++		+	+	4	High risk of bias

Tabrizi et al, 2021 (Tabrizi and Farhangi, 2021)	Cross-sectional	+	+		++		+		5	High risk of bias
Toopchizadeh et al, 2020 (Toopchizadeh et al., 2020)	Cross-sectional	+			++	++	+		6	High risk of bias

Supplemental Table 2. The Jadad Checklist for Randomized Controlled Trials

**Author, publication year**	**Study design**	**Was the study described as randomized (this includes words such as randomly, random, and randomization)?**	**Was the method used to generate the sequence of randomization described and appropriate (table of random numbers, computer-generated, etc)?**	**Was the study described as double blind?**	**Was the method of double blinding described and appropriate (identical placebo, active placebo, dummy, etc)?**	**Was there a description of withdrawals and dropouts?**	**Score**	**Interpretation**
Yucel et al, 2021 (Yucel et al., 2021)	RCT	1	1	0	0	1	3	superior quality
Mousavi-Shirazi-Fard et al, 2021	RCT	1	1	0	0	1	3	superior quality

(Mousavi-Shirazi-Fard et al., 2021)								
